# Germination heterochrony in annual plants of *Salsola* L.: an effective survival strategy in changing environments

**DOI:** 10.1038/s41598-018-23319-0

**Published:** 2018-04-26

**Authors:** Hua F. Liu, Tong Liu, Zhi Q. Han, Ning Luo, Zun C. Liu, Xiao R. Hao

**Affiliations:** 1 College of Life Sciences, Shihezi University, Shihezi, 832003 China; 2 College of Science, Shihezi University, Shihezi, 832003 China

## Abstract

Germination heterochrony refers to germination of seeds dispersed in a single growing season, which is different from delayed germination. We studied two year’s demographic characteristics, characteristics of fruit heteromorphism, the relationship between fruit heteromorphism and germination heterochrony, effects of moisture and temperature on germination characteristics, as well as seed longevity of four annual *Salsola* L. species to analyze the adaptive significance and causes of germination heterochrony. We found that the number of individuals of all populations changed drastically in one year. Approximately 41.6–100% of seedlings germinated in spring died. The number of fruit types varied with interspecies and intraspecies. Despite the wide range of germination temperature of different fruit types (0–35 °C), the germination percentage at 0–15 °C was the highest. When the soil moisture content was 20%, the germination percentage was the highest, reaching 50% within the shortest time. The contrary was the case with the decreasing of soil moisture. The seed longevity of the four species was one year. Fruit heteromorphism had no direct relationship to germination heterochrony. Germination heterochrony was caused by precipitation characteristics and short seed longevity of annual *Salsola* L., which was an effective survival strategy for plant to adapt to the changing environments in arid area.

## Introduction

Since seed germination is one of the most critical stages in the life cycle of plants, the differences in germination time have important effects on plant growth, competitiveness and reproduction, particularly for annuals in arid areas^1–3^. At present, most researches have been focusing on the characteristics of seed germination. However, the research on how germination characteristics impact population maintenance is relatively few^4–7^. Study on the effects of germination characteristics on population maintenance can give a comprehensive understanding of the causes and adaptive significance of germination characteristics.

Germination heterochrony, being different from delayed germination, refers to germination of seeds dispersed in a single growing season (e.g., spring, summer, and autumn), which exhibits the behavior of continuous germination during this period of time. Delayed germination is a typical example of bet-hedging. In terms of delayed germination, a fraction of seeds produced in the previous year fail to germinate completely, remaining in the soil seed bank and ready to germinate in the coming years^8–10^. Many annual plants employ this strategy to adapt to the heterogeneous environment in arid areas. To date, a great number of studies focus on delayed germination, but only a few on germination heterochrony^11–13^.

Many abiotic and biotic factors influence seed germination, and factors such as seed dormancy^14–16^, longevity^17,18^, maternal effects^19,20^, and particularly seed heteromorphism play an important role^21–26^. Seed heteromorphism refers to a phenomenon that a single plant produces different types of seeds or fruits as a strategy of adaptation to the spatial-temporal variability of habitats^27,28^. So far, few studies focus on the relationship between germination heterochrony and seed heteromorphism.

As a member of Chenopodioideae (family Amaranthaceae), *Salsola* L. is mainly distributed in arid areas of Asia, Europe and Africa^29^. Approximately 37 species of *Salsola* exist in China, mainly growing in the Junggar Basin of Xinjiang Uygur Autonomous Region^30,31^. Annual species of *Salsola* are an indicator species of degraded vegetation areas and pioneer species of secondary barren areas, which have important ecological functions in sand fixation. Recently, we observed that annual species of *Salsola* had the ability to germinate at different periods (spring, summer and autumn) in the field environment within a year. No matter how late the seeds started to germinate, most plants germinated in summer and autumn were able to complete their life cycle and produce seeds before winter. All the species exhibited fruit heteromorphism on different parts of the plant. According to recent studies, different time of seed germination was generally associated with seed heteromorphism due to the differences in dormancy breaking and germination requirements^28,32,33^. Therefore, whether the germination heterochrony in *Salsola* L. was caused by fruit heteromorphism requires further study.

Since germination heterochrony and fruit heteromorphism were common in annual *Salsola* L., we hypothesized that (1) germination heterochrony was an adaptive strategy to avoid local population extinction due to the precipitation fluctuation in a single growing season; (2) there was a correlation between fruit heteromorphism and germination heterochrony; (3) the purpose of germination heterochrony was to improve the efficiency of reproduction and reduce investment losses of the offspring, considering that the seed longevity was too short while germination had to occur within a single growing season.

To verify this hypothesis, four annual species of *Salsola* L. were selected and investigated according to the following methods: (a) demographic characteristics were investigated to study population dynamics in a single growing season; (b) morphology and germination characteristics of fruit heteromorphism were analyzed to determine the relationship between fruit heteromorphism and germination heterochrony; (c) effects of moisture and temperatures on germination characteristics were used to test whether germination heterochrony was related to varying environmental condition; (d) seed germination in the field environment, and characteristics of soil seed banks were investigated to study the seed longevity of the four species.

## Results

### Demographic variability of Salsola species

**Figure 1:**
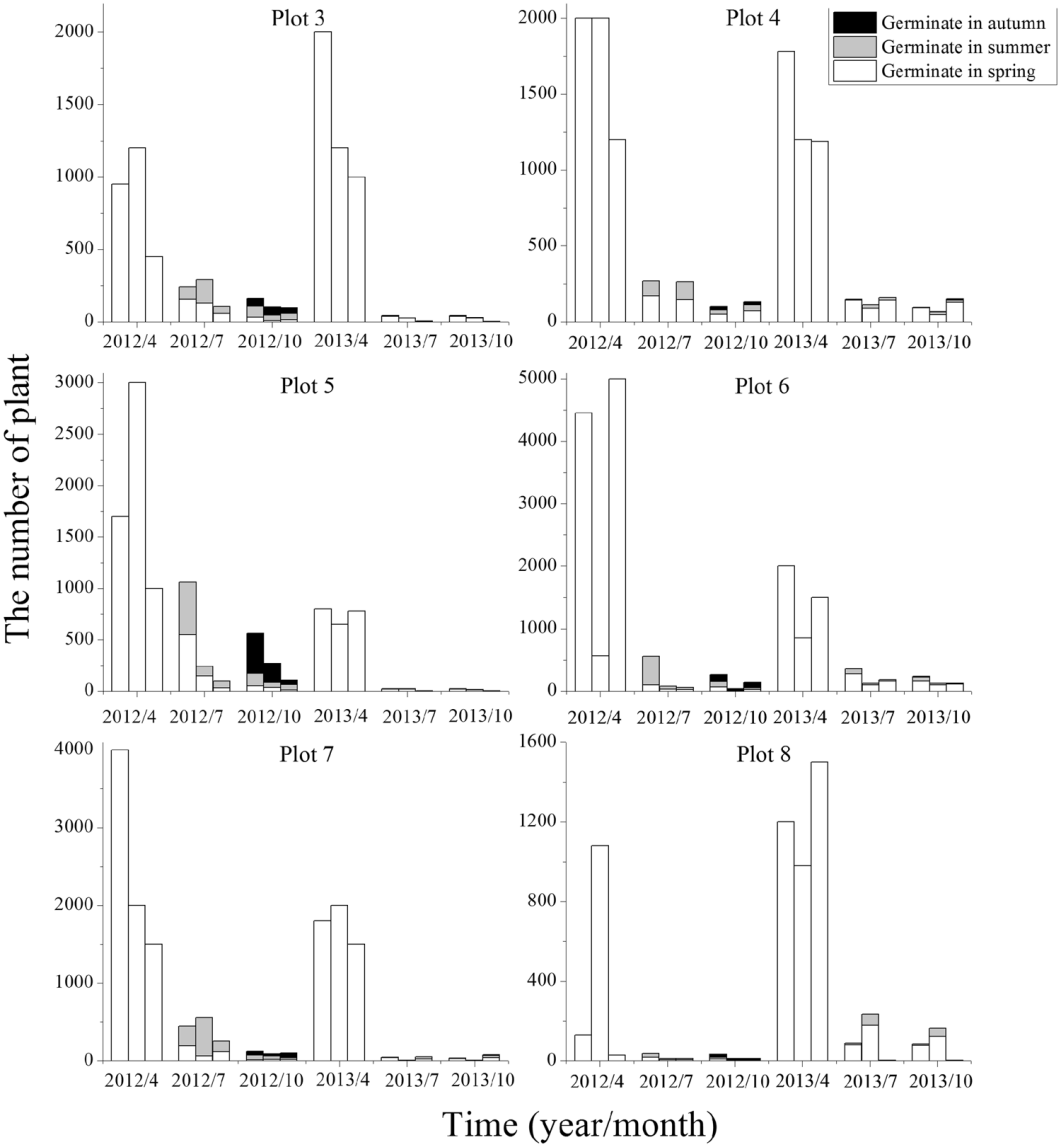
Plant numbers of *S*. *affinis* populations in different periods. Note. Three columns represent three populations of a plot at each time. Refer to Table 1 for the plot number.

*Salsola affinis* populations were observed in three periods (April, July, and October) over two consecutive years. There was a large seasonal fluctuation in terms of the number of individuals within *S*. *affinis* populations (Fig. 1). The number of individuals of the population was generally the largest in spring, with a density of 10–2000 per m^2^. However, there was a great precipitation fluctuation in spring, and seedlings had a mortality rate of 87.4–100.0% in the following summer and autumn. Till the end of the growing season, individuals that germinated in summer and autumn were important to maintain the population. In general, in 2012, individuals that germinated in summer and autumn took up 43.5–93.2% of the number of individuals of the final populations (4.1–66.7% in 2013).

**Figure 2:**
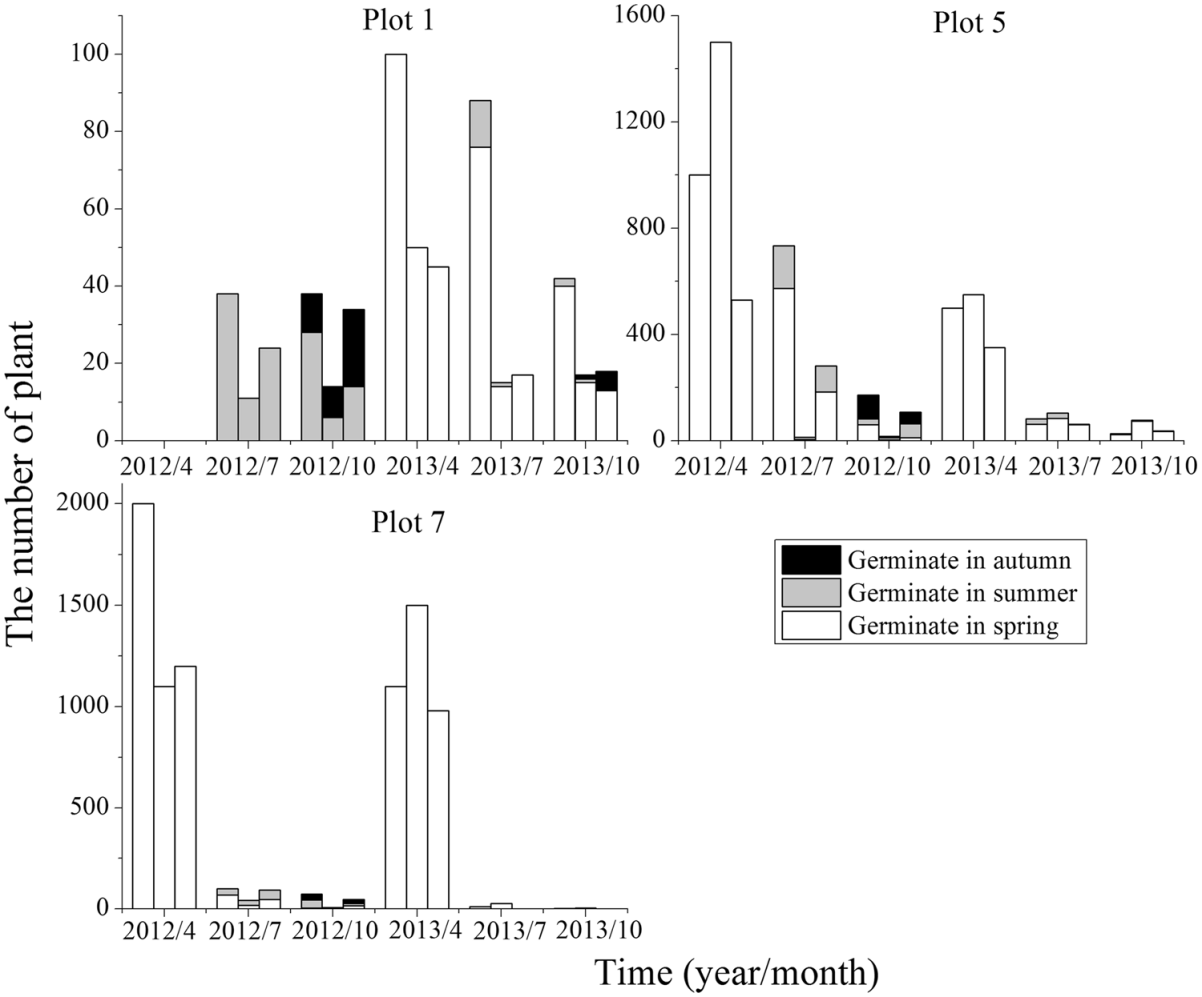
Plant numbers of *S*. *korshinskyi* populations in different periods. Note. Three columns represent three populations of a plot at each time. Refer to Table 1 for the plot number.

In terms of the number of individuals in all populations, *S*. *korshinskyi* was less than *S*. *affinis* (Fig. 2). The drastic seasonal fluctuation of individual number in *S*. *korshinskyi* populations, were similar to that of *S*. *affinis* populations. Seedlings that germinated in summer and autumn were significant for maintaining populations. Due to the lack of spring germination in 2012, individuals that germinated in summer and autumn consisted most of the population in plot 1. In plot 7, no germination was observed in summer and autumn in 2013. This showed that germination time was likely to be affected by environmental variations. In general, seedlings that germinated in spring had a mortality rate of 60.0–100.0% in the following summer and autumn. In 2012, the final populations were composed of 65.1–100.0% of individuals that germinated in both summer and autumn. Except plot 7 in which all plants died, the final populations consisted of 4.8–27.7% individuals that germinated in summer and autumn in 2013.

**Figure 3:**
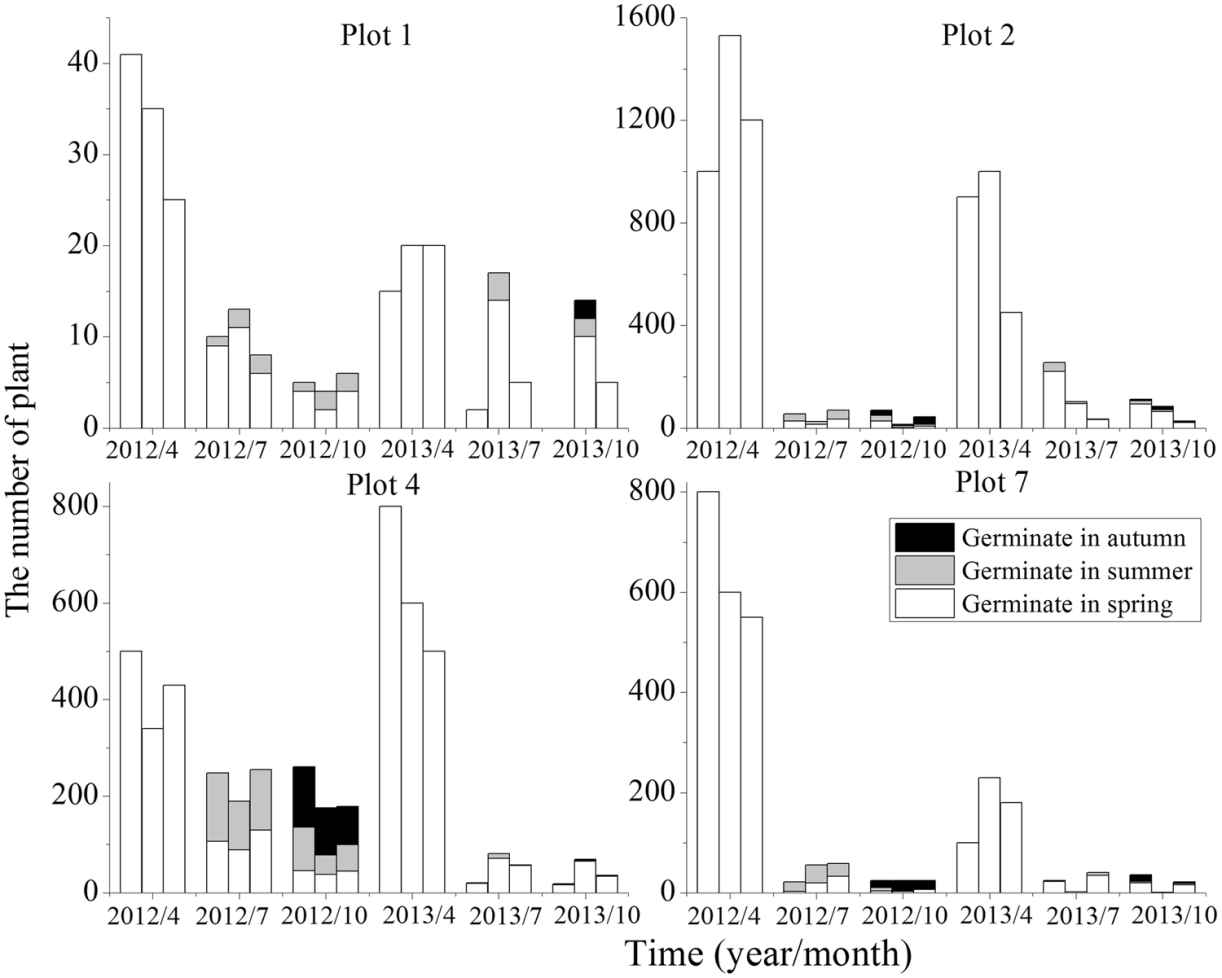
Plant numbers of *S*. *nitraria* populations in different periods. Note. Three columns represent three populations of a plot at each time. Refer to Table 1 for the plot number.

The populations of *S*. *nitraria* were less than those of *S*. *affinis* and *S*. *korshinskyi* in terms of the number of individuals with a range of 15–1500 plants for each population in spring. The number of individuals of *S*. *nitraria* populations exhibited drastic seasonal fluctuation throughout the growing season (Fig. 3). Overall, seedlings that germinated in spring had a mortality rate of 50.0–100.0% in the coming summer and autumn in 2012 and 2013. In addition, towards the end of the growing season, individuals that germinated in summer and autumn were significant for maintaining the populations. In 2013, germination did not occur in summer and autumn in the first and third populations of plot 1 as well as the second population of plot 7. The final populations were composed of 20.0–87.5% individuals that germinated in summer and autumn in 2012, compared with 5.8–44.4% in 2013.

**Figure 4:**
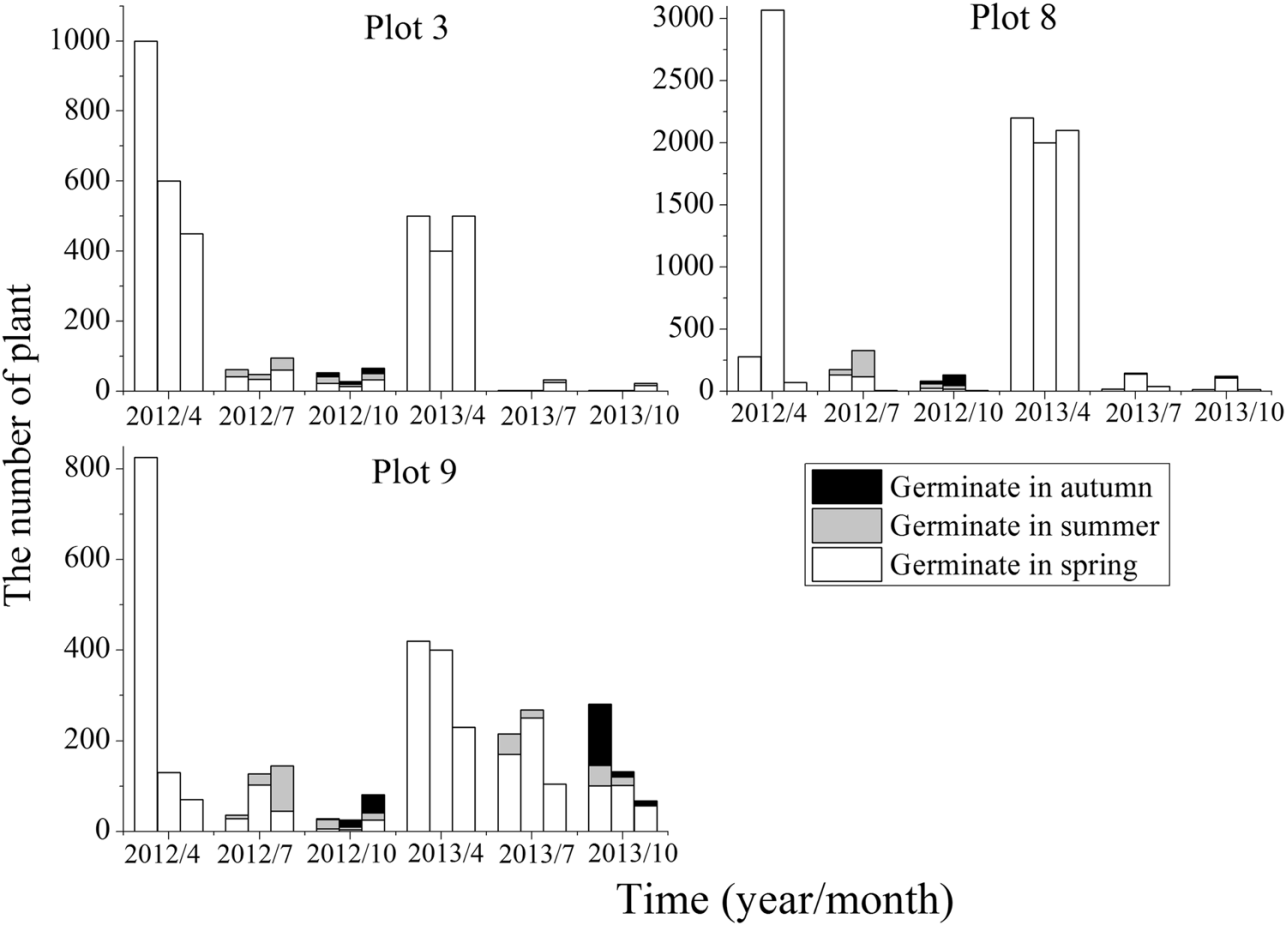
Plant numbers of *S*. *brachiata* populations in different periods. Note. Three columns represent three populations of a plot at each time. Refer to Table 1 for the plot number.

The population size of *S*. *brachiata* also exhibited seasonal fluctuation which was similar to other species (Fig. 4). Seedlings germinated in spring had a mortality rate of 64.2–99.6% in the following summer and autumn. The seedlings that germinated in summer and autumn played a vital role as to the maintenance of populations. In 2013, germination was not observed during summer and autumn in the first and second populations of plot 3, and the first population of plot 8. The final populations were comprised of 50.0–87.1% of individuals that germinated in summer and autumn in 2012, compared with 9.2–64.1% in 2013.

### Characteristics of fruit heteromorphism

Fruit size varied greatly among fruit types within the same species (fruit characteristics are shown in Table 1). Different fruit types, A–D, were used for classification based on bract features, such as appendage size and growth situation (the photograph of fruit type see supplemental files). *S*. *brachiata* had only three fruit types (A, B, and D) in three plots (nine populations). Nevertheless, *S*. *affinis*, *S*. *korshinskyi*, and *S*. *nitraria* had four types (A–D) among some populations; or A, B, and D in other populations. Fruit heteromorphism characteristics of the four species revealed significant differences in the fruit wing size among the four types in the same species. The size followed the type order of A > B > C > D. However, there was no difference in fruit mass.

**Table 1:** Characteristics of fruit types (FT) A, B, C, and D (mean ± s.e.).

Species	FT	Wings	Orientation of the seed	Diameter (cm)	Hundred-fruit mass(g)
*S*. *affinis*	A	Long	Horizontal	0.650 ± 0.151a	0.3564 ± 0.1708a
	B	Short	Horizontal	0.413 ± 0.120b	0.2453 ± 0.1082b
	C	Bulge	Horizontal	0.309 ± 0.073c	0.1991 ± 0.0422b
	D	Absent	Vertical	0.194 ± 0.040d	0.2048 ± 0.0782b
*S*. *korshinskyi*	A	Long	Horizontal	1.140 ± 0.205a	0.6877 ± 0.0951a
	B	Short	Horizontal	0.728 ± 0.125b	0.4470 ± 0.0838b
	C	Bulge	Horizontal	0.349 ± 0.070c	0.3440 ± 0.0932b
	D	Absent	Vertical	0.199 ± 0.047d	0.3660 ± 0.1402b
*S*. *nitraria*	A	Long	Horizontal	0.580 ± 0.079a	0.1789 ± 0.0560a
	B	Short	Horizontal	0.365 ± 0.066b	0.1215 ± 0.0456a
	C	Bulge	Horizontal	0.330 ± 0.055b	0.1870 ± 0.0070a
	D	Absent	Sideling	0.176 ± 0.037c	0.1703 ± 0.1961a
*S*. *brachiata*	A	Long	Horizontal	0.890 ± 0.146a	0.6797 ± 0.1079a
	B	Short	Horizontal	0.520 ± 0.153b	0.4441 ± 0.1240b
	D	Absent	Vertical	0.236 ± 0.052c	0.2932 ± 0.0696c

Note. Different lowercase letters (a, b, c, and d) within the same column indicate significant differences at *p* < 0.05 in the characteristics of fruit types in the same species.

### Effect of temperature on germination of fruit types

**Figure 5:**
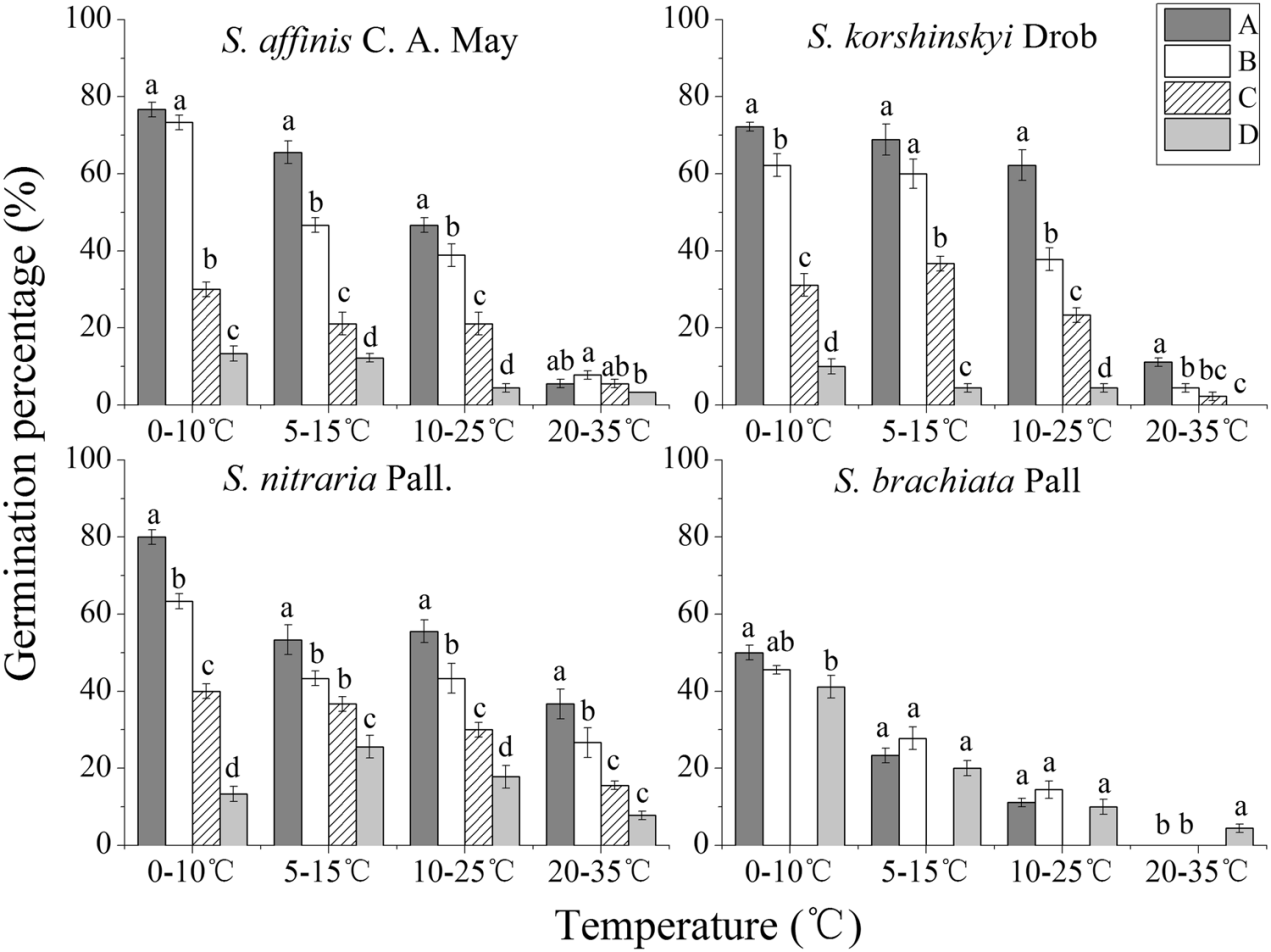
Final germination percentage of the different fruit types of four *Salsola* species at different alternating temperatures. Note. Different lowercase letters indicate significance difference at p < 0.05 among the fruit types at the same temperature.

With different temperatures, different fruit types in the four species had varying germination percentage (Fig. 5). The germination percentage of type A fruits of *S*. *affinis*, *S*. *korshinskyi*, *S*. *nitraria*, and *S*. *brachiata* were higher than those of other three fruit types at 0/10 °C, 5/15 °C, and 10/25 °C. All fruit types of *S*. *brachiata* had the highest germination percentage at 0/10 °C. The two-way ANOVA showed that the responses to different temperatures varied among fruit types in each species, which could be detected as a significant interaction effect (temperature × fruit-type). The germination percentage of *S*. *affinis*, *S*. *korshinskyi*, and *S*. *nitraria* were significantly affected by fruit type, temperature, and interaction. But the difference in germination percentage of *S*. *brachiata* was fruit-type independent (Table 2). Overall, germination percentage of all fruit types in four species was decreased with temperature increasing, and four species had the highest percentage at lower temperatures (0–15 °C). Thus, the germination of different fruit types had no special response to temperature variations, which implied that temperature was not the cause of large seasonal fluctuation of individual numbers within a population.

**Table 2:** Analysis of variance for effects of temperature (T), fruit type (FT) and their interaction on the germination percentage of four species.

Source of variation	*S. affinis*		*S. korshinskyi*		*S. nitraria*		*S. brachiata*	
	df	F	df	F	df	F	df	F
T	3	331.523^**^	3	234.468^**^	3	73.348^**^	3	316.475^**^
FT	3	359.967^**^	3	318.144^**^	3	169.986^**^	2	2.939
T × FT	9	44.952^**^	9	26.000^**^	9	9.242^**^	6	3.626^*^

Note. ^*^indicate significance at the 5% level, ^**^indicate significance at the 1% level.

### Effect of soil moisture content and temperature on germination of type A and B fruits

Standard factorial experiments revealed that, four species had varying germination characteristics with different soil moisture content at different temperatures (Table 3). The germination percentage and germination rate of type A and B fruits of all species were the highest at the lowest temperature (0–10 °C) and the highest soil moisture content (20%), which decreased with the drop of soil moisture content or the rise of the temperature. Furthermore, the two-way ANOVA analysis showed that soil moisture content and temperature had considerable influences on the number and the rate of germinated seeds in the four species. But the interaction between the two environmental factors had a significant impact on those of *S*. *affinis* and *S*. *brachiata* (Table 4). The result indicated that the amount of local precipitation could affect both the number of seeds germinated and the germination rate, which jointly determined the survival rate of seedlings.

**Table 3:** Effect of soil moisture content on germination of fruits type (FT) A and B at variable temperatures (T).

Species	FT	T	Soil moisture content					
			5%	10%	20%	5%	10%	20%
			Germination percentage (%)			Germination rate (%)		
*SA*	A	0/10 °C	35.56 ± 4.01c	56.67 ± 5.09b	85.56 ± 2.94a	11.02 ± 2.01b	25.56 ± 4.84a	37.78 ± 4.01a
		10/25 °C	32.22 ± 2.94b	40.00 ± 3.85b	60.00 ± 3.85a	7.04 ± 0.67b	9.81 ± 1.77b	22.96 ± 1.96a
		20/35 °C	7.78 ± 1.11c	30.00 ± 1.92b	45.56 ± 2.22a	1.83 ± 0.35c	7.50 ± 0.48b	12.59 ± 1.04a
	B	0/10 °C	33.33 ± 1.92c	51.11 ± 2.94b	68.89 ± 2.94a	7.83 ± 0.95b	19.81 ± 2.77a	26.48 ± 2.61a
		10/25 °C	22.22 ± 2.94b	24.44 ± 2.94b	57.78 ± 4.84a	5.19 ± 0.27c	9.26 ± 0.74b	14.44 ± 1.21a
		20/35 °C	4.44 ± 1.11c	21.11 ± 2.94b	37.78 ± 2.94a	1.39 ± 0.42b	7.04 ± 0.98a	9.44 ± 0.73a
*SK*	A	0/10 °C	33.33 ± 1.92c	51.11 ± 2.94b	72.22 ± 4.01a	9.17 ± 0.48c	15.65 ± 1.84b	24.07 ± 1.34a
		10/25 °C	27.78 ± 2.94c	43.33 ± 1.92b	64.44 ± 2.94a	6.94 ± 0.73c	10.83 ± 0.48b	16.11 ± 0.73a
		20/35 °C	18.89 ± 2.94c	32.22 ± 2.94b	47.78 ± 4.01a	4.72 ± 0.73b	10.74 ± 0.98a	14.54 ± 1.64a
	B	0/10 °C	23.33 ± 1.92c	41.11 ± 4.01b	62.22 ± 2.94a	7.22 ± 1.16c	13.7 ± 1.34b	18.89 ± 1.28a
		10/25 °C	23.33 ± 3.85b	28.89 ± 2.94b	56.67 ± 3.85a	5.83 ± 0.96b	9.63 ± 0.98ab	13.22 ± 1.35a
		20/35 °C	13.33 ± 1.92b	26.67 ± 1.92a	32.22 ± 4.01a	3.33 ± 0.48b	8.15 ± 0.98a	9.63 ± 0.37a
*SN*	A	0/10 °C	55.56 ± 2.94b	66.67 ± 3.85ab	77.78 ± 4.01a	21.30 ± 1.88b	33.33 ± 1.92a	34.26 ± 3.55a
		10/25 °C	30.00 ± 3.85b	54.44 ± 2.94a	64.44 ± 4.01a	15.00 ± 1.92b	27.22 ± 1.47a	28.33 ± 2.89a
		20/35 °C	21.11 ± 2.94c	41.11 ± 4.01b	57.78 ± 2.94a	9.63 ± 2.25b	18.15 ± 2.67ab	25.74 ± 3.72a
	B	0/10 °C	51.11 ± 2.94a	52.22 ± 2.94a	55.56 ± 4.44a	15.65 ± 1.84b	17.41 ± 0.98b	27.78 ± 2.22a
		10/25 °C	24.44 ± 2.94b	40.00 ± 3.85a	50.00 ± 1.92a	9.26 ± 0.74c	13.33 ± 1.28b	16.67 ± 0.64a
		20/35 °C	20.00 ± 1.92b	40.00 ± 1.92a	47.78 ± 2.94a	7.59 ± 0.49c	13.33 ± 0.64b	15.93 ± 0.98a
*SB*	A	0/10 °C	25.56 ± 2.94c	38.89 ± 2.94b	70.00 ± 1.92a	8.80 ± 0.72b	13.70 ± 1.96ab	25.46 ± 5.77a
		10/25 °C	15.56 ± 1.11c	32.22 ± 2.94b	47.78 ± 4.01a	4.44 ± 0.64b	9.11 ± 1.10b	23.89 ± 2.00a
		20/35 °C	10.00 ± 1.92b	25.56 ± 2.94a	21.11 ± 2.94a	3.33 ± 0.64a	7.22 ± 1.47a	6.57 ± 1.36a
	B	0/10 °C	22.22 ± 2.94c	34.44 ± 2.94b	62.22 ± 4.01a	7.41 ± 0.98b	11.48 ± 0.98b	20.74 ± 1.34a
		10/25 °C	16.67 ± 1.92b	24.44 ± 2.94b	43.33 ± 1.92a	5.09 ± 0.79b	7.31 ± 0.33b	16.67 ± 1.7a
		20/35 °C	7.78 ± 1.11b	16.67 ± 1.92a	16.67 ± 1.92a	2.96 ± 0.37b	5.00 ± 0.32ab	7.41 ± 1.34a

Note. Different lowercase letters (a, b, and c) within the same row indicate significance at *p* < 0.05 among the different soil moisture content at the same temperature. *SA*, *SK*, *SN*, *SB* represent *S*. *affinis*, *S*. *korshinskyi*, *S*. *nitraria*, *S*. *brachiata* respectively.

**Table 4:** Two-way ANOVA of effects of soil moisture content (SMC) and temperature (T) on germination percentage and germination rate of type A and B fruits in four species.

Fruit type	Dependent variable	Source of variation	*S. affinis*		*S. korshinskyi*		*S. nitraria*		*S. brachiata*	
			df	F	df	F	df	F	df	F
Type A	Germination percentage	SMC	2	101.85^**^	2	99.28^**^	2	58.86^**^	2	85.15^**^
		T	2	67.76^**^	2	31.00^**^	2	43.82^**^	2	66.82^**^
		SMC × T	4	3.56^*^	4	0.78	4	1.59	4	13.36^**^
	Germination rate	SMC	2	41.17^**^	2	78.95^**^	2	24.69^**^	2	25.18^**^
		T	2	40.68^**^	2	27.35^**^	2	15.63^**^	2	15.46^**^
		SMC × T	4	3.54^*^	4	2.39	4	0.54	4	4.48^*^
Type B	Germination percentage	SMC	2	104.97^**^	2	69.96^**^	2	31.95^**^	2	75.15^**^
		T	2	75.89^**^	2	25.66^**^	2	28.86^**^	2	78.53^**^
		SMC × T	4	3.06^*^	4	4.58^*^	4	5.14^**^	4	11.64^**^
	Germination rate	SMC	2	51.40^**^	2	49.81^**^	2	43.48^**^	2	73.51^**^
		T	2	54.15^**^	2	27.11^**^	2	38.69^**^	2	47.54^**^
		SMC × T	4	4.37^*^	4	2.04	4	3.17^*^	4	6.30^*^

Note. ^*^indicate significance at the 5% level, ^**^indicate significance at the 1% level.

### Characteristics of soil seed banks

Results from the germination experiment under natural conditions indicated that different fruit types of the four species had relatively higher germination percentage (Table 5). In autumn, the germination of type A fruits of the four species in mesh nylon bags reached 49.11–68.10%, with 42.13– 60.11% for type B, whereas those of types C and D were relatively lower. Based on seed germination experiments for the seed bank, the results showed that no seedlings emerged with sufficient water in plastic plates under indoor condition. Meanwhile, the results of sieving experiments indicated that few fruits (type C and mostly type D) in all soil samples passed through a 0.8-mm mesh screen. In addition, the experiment of the seed germination in the field environment confirmed that the seed bank had relatively fewer seeds left one year later. Finally, the tetrazolium testing of non-germinated seeds in the laboratory showed that all the remaining seeds had lost viability, which implied that the soil seed bank was transient.

**Table 5:** Germination percentage of different fruits in nylon bags in autumn.

Species	Types of fruit	Germination percentage (%)
*S*. *affinis*	A	67.09 ± 2.85a
	B	60.11 ± 2.16a
	C	34.28 ± 2.42b
	D	18.48 ± 0.88c
*S*. *korshinskyi*	A	66.33 ± 3.49a
	B	56.79 ± 1.85b
	C	30.82 ± 2.07c
	D	10.60 ± 1.27d
*S*. *nitraria*	A	68.10 ± 2.25a
	B	55.46 ± 3.03b
	C	39.01 ± 3.21c
	D	12.89 ± 1.16d
*S*. *brachiata*	A	49.11 ± 1.87a
	B	42.13 ± 1.24b
	D	19.05 ± 3.26c

Note. Different lowercase letters (a, b, c, and d) within the same column indicate significant differences at *p* < 0.05 in the characteristics of fruit types in the same species.

## Discussion

Rapid growth rate, high intrinsic rate of increase, precocity, and small in size consist the main evolutionary tendency of annual plants. Annual *Salsola* L. generally has a relatively longer life cycle from seed germination in spring to fruit ripening in late autumn. According to Grime’s theory^34^, they belong to the stress-ruderal (SR) species. Along with SR, germination heterochrony is also significant to annual *Salsola* L. in arid areas. With a two-year field investigation and laboratory experimentation, the characteristics and causes for germination heterochrony were determined and the adaptation significance for population maintenance under changing environments was verified.

### Germination heterochrony: an effective strategy for annual *Salsola* L. to avoid local population extinction in a single growing season

The population dynamics of plants is resulted from interaction between individual survival and environmental conditions^35^. Population characteristics directly indicate the status of survival and reproduction in a population, and the struggle between the plant population and environmental conditions. In arid areas, the time, intensity, amount and patterns of precipitation are highly variable^36,37^, which impose obvious effects on plant growth. In fact, the relatively high mortality rate of seedlings in these areas causes the fluctuation of population sizes. Until now, there are few studies on the relationship between germination strategy and the fluctuation of plant populations^4–7^. The two-year study found that the number of individuals of *Salsola* L. populations changed drastically over a single growing season. The time of the first seed germination fluctuated between March and July, subsequently accompanied by continuous seed germination and deaths of large numbers of seedlings in population. Most of the population had thousands of individuals in spring, but only a small collection of individuals (0–59.4%) survived till the flowering and the fruiting stages.

It was found that both endogenous and exogenous factors contributed to temporal variation in the number of plant populations. Large population density and precipitation fluctuation were usually considered the cause of individual death. Since resources within a given area were limited, some individuals died with the increasing density of population. Obviously, plants cannot grow when the resources were running out. But in our study, both high and low-density populations showed a high mortality rate. For instance, the spring seedling of *S*. *affinis* was 595 plants per m^2^ in the second population of plot 5 and thirteen plants per m^2^ in the first population of plot 8, both had a high mortality rate (97.1% and 86.2%). In fact, population dynamics of the four *Salsola* L. was not caused by population density as a whole. However, towards the end of the growing season, individuals that germinated in summer and autumn accounted for more than half of the population and even reached 100%. Regardless of how late the germination time was, the plants could complete their life cycle and produce seeds before winter, which was a special reproductive characteristic of annual *Salsola*. Therefore, germination heterochrony served to regulate population dynamics and maintain the population in a more efficient way.

For annual plants inhabiting environments where reproductive success is highly variable and unpredictable, seed germination usually adopts a bet-hedging strategy^38^. By spreading seed germination over several years, desert annuals may reduce variation in fitness, and buffer the risk of extinction in unfavourable years with strategies such as delayed germination^39,40^. By contrast, germination heterochrony of *Salsola* L. is more beneficial to the development and growth of population. Since annual *Salsola* L. has a short seed longevity (about one year), the seed is forced to germinate in a single growing season, making full use of the limited precipitation in order to reduce investment losses of the offspring, particularly under the pressure of great precipitation fluctuation. As proven by the field investigation and laboratory experiment, the low precipitation amount in a single time and low germination percentage led to continuous germination of the remaining seeds with subsequent precipitation. Provided that all the seeds germinate at one time, there exists the risk of population extinction under this circumstance. In this way, the number of individuals within a population was maintained and the continuity of population was ensured. Therefore, germination heterochrony is a special bet-hedging strategy resulted from the germination characteristics, low precipitation amount in a single time and great precipitation fluctuation. Through the investigation of associated species, the results showed that there were only a few species of herbaceous plants (*Calligonum leucocladum*, *Ceratocarpus lateens*, *Salsola praecox*), mainly arbors and shrubs (*Calligonum leucocladum*, *Haloxylon ammodendron*, *Halogeton glomeratus*), could survive extremely poor environmental conditions. However, we found that annual *Salsola* L. was the dominant species which outnumbered other plants in the region. Thus, the wide distribution of *Salsola* L. in the Junggar Basin might be related to the high efficiency of germination heterochrony.

### Fruit heteromorphism has no direct relation with germination heterochrony in *Salsola* L

Fruit heteromorphism is common in desert plants, which is of great ecological and evolutionary significance in escaping the negative effects of crowding, reducing sibling competition, and adapting to the heterogeneous environments with a bet-hedging strategy^28,41^. It may also represent a mixed strategy, reducing temporal variance in fitness^25,26,42^. In this study, annual *Salsola* L. had three (A, B, D) or four (A, B, C, D) types of fruits in different populations of the same species with significant intraspecific differences in fruit size and bract presence. In the past, most researches tended to focus on a single population, instead of the difference of fruit heteromorphism among populations. *S*. *affinis* and *S*. *korshinskyi* had three types of fruit^43,44^, while *S*. *brachiata* had four types of fruit^45^. Our classification differed from that of fruit (utricles) types in previous studies. With comparison of fruit types of different plots, we found that the number of fruit types of species was different among plots, which might be caused by the variation in the environment.

**Figure 6:**
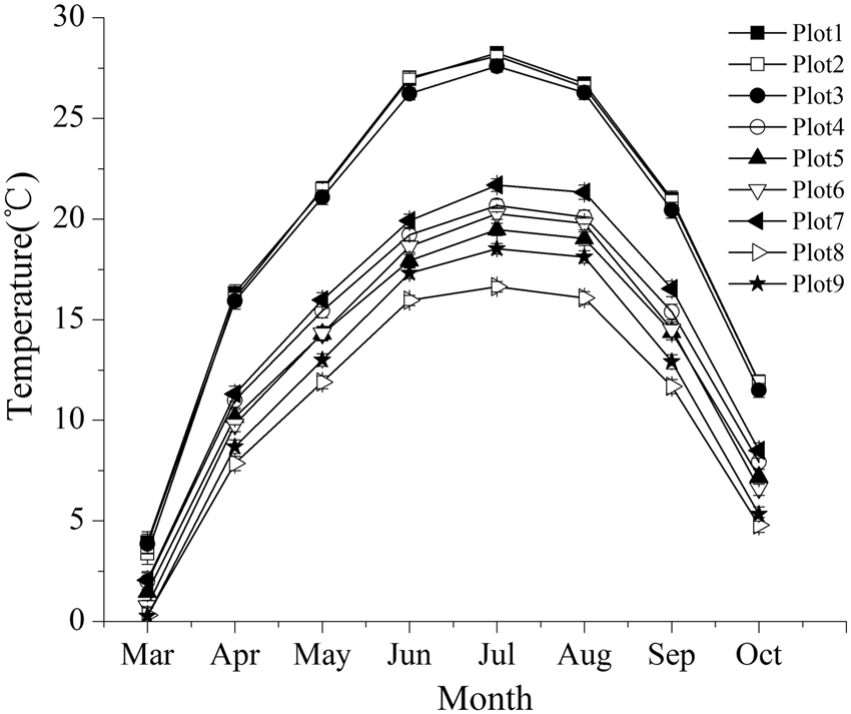
The average monthly temperature of nine plots in 2012 and 2013.

Fruit heteromorphism is frequently accompanied by different germination behaviors and dispersal capacity. Different fruit types of *S*. *affinis*, *S*. *brachiata* and *S*. *korshinskyi* represent two mechanisms of dispersal and germination. They are ‘escape’ dispersal strategy and ‘opportunistic’ germination strategy, ‘protection’ dispersal strategy and ‘conservative’ germination strategy^43–45^. Our results are similar to previous studies mentioned in this paper. We observed that germination percentage was fruit-type dependent (fruit type × temperature) through two-way ANOVA. The decline in germination percentage of type A and B was more dramatic for than that of type D. This result revealed that evolutionary significance of the type D: it contributes to persistence because of its tolerance of high temperature. However, it was particularly noteworthy that the germination percentage of fruit types A–D all decreased with temperature increasing, while germination of different fruit types was not specific for temperature. Regardless of the fruit type, the highest germination percentage were at the lower temperature (0–15 °C), which was consistent with germination situation in field actual temperature condition (see Fig. 6), showing that seeds tended to germinate in early spring. Meanwhile, by investigating demographic characteristics over two years (see Figs 1–4), the results demonstrated that the number of individuals of annual *Salsola* L. population was the highest in spring, which meant that the number of seed germination was determined by temperature. As shown by the results above, regardless of the fruit type, the earlier the seed germinates, the greater the mortality risk of seedling will be. But the individuals with earlier germination will have a priority to resources available, which enhances its competitiveness within a longer growing period, producing a greater number of fruits. Therefore, fruit heteromorphism has no direct relationship with germination heterochrony.

### Low amount of precipitation in a single time, great precipitation fluctuation and short seed longevity: the causes of germination heterochrony

In arid systems, precipitation is a major environmental factor which affects seed germination. In the Junggar Basin, the precipitation is highly variable, with low precipitation (<5 mm) accounting for 87.5% of the total annual rainfall frequency, whereas precipitation more than 10 mm accounts for only 4.3%^46^, and a monthly precipitation coefficient of variation showed a range of 47.12–74.62%. According to the fluctuation of precipitation and population, the high monthly precipitation coefficient of variation for each plot could explain why germination time and demography of the four species were seriously affected by seasonal precipitation changes in natural habitats. At the same time, even though the precipitation was highly variable, they germinated at any time of growing season due to their wide range of temperature tolerance if the moisture condition was suitable. So, it led to continuous seed germination from spring to autumn. According to the research on effects of soil moisture content and temperature on germination of type A and B fruits, we found that the increasing of the moisture could significantly promote the total germination percentage and rate of the four species, however, opposite results were obtained with moisture reduction. The findings implied that the high germination rate served to ensure an adequate growing time with limited soil moisture while completing seedling establishment and reducing its mortality rate. However, the low germination rate meant a longer time for germination. With low air humidity and fast soil water evaporation in arid area, a large number of seedlings were supposed to die because of lack of water, especially when there was no constant precipitation. Thus, violent precipitation fluctuation and low precipitation amount in a single time were decisive to population fluctuation in annual *Salsola* L.

Meanwhile, we found that the soil seed bank of the four species was transient. The indoor and field experiments proved that fruit types A and B of all tested species had a higher germination percentage while the lower germination percentage of fruit types C and D might form the seed bank. With seed bank experiment, we found that the soil seed bank of the four annual species only included a few fruits (type C and D, mostly type D), which indicated their soil seed bank was transient. The finding was similar to Chenopodiaceae plants *Salicornia europaea*^41^ and *Atriplex triangularis*^47^. But tetrazolium testing showed that the remaining seeds lost viability, revealing that the seed longevity of annual *Salsola* L. was short and seeds needed to germinate in a single growing season to reduce reproductive investment losses of previous generation to seed production. Thus, to maintain the greatest growth benefits within the year, short seed longevity was the main biotic reason for germination heterochrony. In addition, due to the low amount of precipitation in a single time, it is impossible for all the seeds to germinate at one time. Thus, a continuous germination will occur with the constant precipitation fluctuation to maintain the number of individuals of the population, and fitness of population to the greatest extent. Therefore, the low precipitation amount in a single time, and significant precipitation fluctuation were confirmed as the major environmental reason for germination heterochrony.

To date, there are a few studies on germination heterochrony^11–13^. However, our research was based on field investigation over the past several years, which confirmed that germination heterochrony was continuous seed germination within a single growing season in terms of population demography. Regardless of how late the germination time was, the plant completed their life cycle and produce seeds before winter, which had not been reported by previous researchers.

## Materials and Methods

### Study area

The study focuses on areas in the semi-closed Junggar Basin (34°09′–49°08′ N and 73°25′–96°24′ E). The Gurbantünggut Desert lies in the center of the Junggar Basin. The meteorological data of Junggar Basin shows a large precipitation fluctuation with rain fall amount greater than 5 mm, accounting for 87.5% of the total annual rainfall. The mean annual temperature of the area is 6.8 °C, and the extreme temperatures of the coldest (January) and hottest (July) months are −32.8 and 40.5 °C, respectively. The annual potential evaporation is over 2000 mm^48^.

Vegetation in the Junggar Basin mainly consists of xerophytes and super xerophytic shrubs. Annuals are widely distributed with simple community structure. The plant composition is dominated by Chenopodiaceae, and other major families including Brassicaceae, Asteraceae, Leguminosae, and Graminae^49,50^.

### Study species and study site

In this study, four annual species of the genus *Salsola* are used: *Salsola affinis* C. A. Mey, *Salsola korshinskyi* Drob., and *Salsola brachiata* Pall. These species are widely distributed in the Junggar Basin^49^. *S*. *affinis*, an inland salt desert annual, is highly tolerant in aridity and saline-alkaline conditions. *S*. *korshinskyi* grows in saline soils and has a strong drought and salt-tolerance. *S*. *nitraria* mainly distributes on the edge of the Gurbantünggut Desert. *S*. *brachiata* grows primarily in gravel deserts and foothills. Annual *Salsola* L. has hermaphrodite flowers, with the ability of self-pollination, which have 5 stamens and 2 stigmas relying on wind-and insect-pollination. The fruiting season lasts from September to October^49^.

Our research covered the southern margin of the Junggar Basin. A total of 13 sites were designed, with 60 km between each site. Of the 13 sites, only nine were in the annual *Salsola* L. distribution area and permanently marked plots were established along the edge of the hinterlands of Junggar Basin, including in the desert plain, saline soils, foothills, and sand desert areas. Each plot had three populations (quadrats) for one species if it occurred on the site. Because the spatial patterns of all populations were clumped distributions, quadrat area was determined by the natural population area (typically 3–10 m^2^). The linear distance between quadrats was about 10–50 m in each plot. In total, there were 48 quadrats across the nine plots. The main accompanying plants in the quadrats were shrubs, and resource competition was mainly from intraspecific competition. A detailed description of habitat type, associated species, and coordinates for each plot was found in Table 6.

**Table 6:** Habitat characteristics of the distribution of the four *Salsola* L. species.

Plots	Species	Elevation(m)	Habitat	Main associated species	Coordinates	Precipitation/mm	
						Mean	CV
1	*S*. *korshinskyi S*.*nitraria*	258	Saline soils	*Calligonum leucocladum*; *Halogeton glomeratus*	N45°28′ E85°38′	24.07	47.12
2	*S*.*nitraria*	276	Inter-dune lowland	*Ceratocarpus lateens*; *Haloxylon ammodendron*	N45°10′ E86°15′	30.55	54.73
3	*S*. *affinis S*. *brachiata*	276	Plain desert	*Reaumuria soongorica*; *Haloxylon ammodendron*; *Salsola praecox*	N45°14′ E84°45′	55.93	51.51
4	*S*. *affinis S*.*nitraria*,	328	Gravel desert	*Haloxylon ammodendron*; *Halogeton glomeratus*	N44°54′ E82°25′	296.30	60.26
5	*S*. *affinis S*. *korshinskyi*,	489	Gravel desert	*Salsola praecox*; *Haloxylon ammodendron*	N44°42′ E82°05′	374.91	66.05
6	*S*. *affinis*	491	Gravel desert	*Calligonum leucocladum*; *Haloxylon ammodendron*	N44°25′ E84°00′	268.18	47.12
7	*S*. *affinis S*. *korshinskyi S*.*nitraria*	514	Sand desert edge	*Haloxylon ammodendron*; *Ceratocarpus arenarius*	N44°28′ E82°54′	283.32	74.62
8	*S*. *affinis S*. *brachiata*	719	Foothills of mountain	*Halogeton glomeratus*; *Haloxylon ammodendron*	N44°03′ E86°31′	602.84	53.78
9	*S*. *brachiata*	919	Foothills of mountain	*Halogeton glomeratus*; *Salsola praecox*; *Atriplex dimorphostegia*	N44°16′ E84°52′	454.36	51.03

Note. Plots 1–9 represent nine permanently marked plots respectively. Mean represents mean annual precipitation (average for the two years from March to October), CV represents monthly precipitation coefficient of variation (average for the two years from March to October).

### Meteorological data

To understand the precipitation characteristics of the researching area, the meteorological data needs to be analyzed. However, there are no weather stations near the plots in the hinterland of the Junggar Basin. Therefore, reanalysis data was applied to analyze precipitation characteristics in the region. Previous studies had proven this method to be highly accurate^51^. Consequently ERA-Interim forecast precipitation data provided by European Centre for Medium Range Weather Forecasts was referred in this study^52^. Focusing on a 0.125° latitude longitude grid for March to October in 2012 and 2013, we calculated monthly precipitation coefficient of variation (CV), mean annual precipitation and monthly temperature initialized at 00 Universal Time Coordinated (UTC). To accomplish this, we applied 12 hour forecast precipitation data (00, 12 UTC) initialized at 00 UTC, and mean monthly temperature calculated by applying 6 hours forecast 2 m temperature data (00, 6UTC, 12 UTC, 18UTC) initialized at 00 UTC. The precipitation and temperature data from March to October and the growing season of the studied plots are shown in Table 6 and Fig. 6.

### Demographic variability

In 2012 and 2013, demographic characteristics of natural populations were investigated within quadrats (natural population area, typically 3–10 m^2^). Surveys clearly coincided with the identification of three main germination stages: spring (March–April), summer (June–July), and autumn (September–October). All individuals in each quadrat were mapped and new germination and mortality were recorded in April, July, and October. Marked plants were obtained from 48 quadrats in nine plots. The plot numbers of the four species differed in the various environments: there were 18 quadrats of *S. affinis*, nine of *S. korshinskyi*, 12 of *S. nitraria*, and nine of *S. brachiata*. Each plot had at least three quadrats for the existing species. Results gave detailed demographic data for each species over a two years’ period, including population dynamics and phenological characteristics.

### Fruit morphology

At maturity stage (October 2012), plants of the four species were collected from around quadrats in the nine plots. According to the survey, there were three main germination periods of individuals: spring, summer, and early autumn. According to the different germination periods, we observed variation in plant size. Ten plants of each species that germinated in different seasons in each plot were randomly sampled to measure morphological characteristics of their fruit. Thus, 30 plants of each species were sampled in each plot. The samples were taken to the laboratory for seed collection and storage.

For classification of fruit types, the samples collected were used to observe wings, fruit shape and color, and location of fruit on the plant. The hundred-fruit mass and fruit diameter of each species were determined by the average of that of all plots. The mean hundred-fruit mass of each fruit type was determined by weighing three replicates of 100 fruits of each morphotype using an electronic balance (to 0.0001 g). Mean fruit diameter of each fruit type in a plot was calculated from measurements of 10 fruits with a Vernier caliper (to 0.01 mm).

### Effect of temperature on germination

The influence of temperature on germination behavior of Salsola fruits was used to determine the relationship between germination heterochrony and fruit heteromorphism in different seasons. In the final survey, inflorescences were shaken manually to free the fresh fruits from the 48 quadrats, and were stored for 6 months in plastic bags in a ventilated room with a temperature of 10–18 °C and relative humidity of 15–20% before the experiment. Thirty fruits of each fruit type in all species were randomly selected. The germination experiments were repeated three times, with 30 fruits per treatment. For germination, the fruits of each morphotype were placed in 9-cm-diameter Petri dishes on two layers of filter paper (pH = 7) moistened with 10 mL of distilled water. The Petri dishes were incubated at variable temperatures. The following thermoperiods represented the mean daily minimum and maximum temperatures per month in the study area during the growing season: 0/10 °C (early spring), 5/15 °C (spring), 10/25 °C (early summer and autumn), and 20/35 °C (summer). The emergence of the radicle marked germination. Fruit germination percentage was monitored every 24 hours for 21 days. Germination percentage was calculated as follows:

1 $${\rm{Germination}}\,{\rm{percentage}}={\rm{Number}}\,{\rm{of}}\,{\rm{fruits}}\,{\rm{germinated}}/{\rm{Total}}\,{\rm{number}}\,{\rm{of}}\,{\rm{fruits}}\times 100 \% $$

### Effect of soil moisture content and temperature on germination of type A and B fruits

To clarify the effect of precipitation fluctuation relating on the germination heterochrony of four species in different seasons, a split- plot experiment was conducted in artificial climate chambers. The main factor was temperature (i.e., 0–10 °C, 10/25 °C and 20–35 °C) representing different seasons (early spring, early summer, autumn and summer respectively). The sub-factor was soil moisture content (i.e., 5%, 10% and 20%) representing different amount of precipitation. The seed germination and growth of annual plants in arid areas mainly rely on precipitation. In this experiment, the number of type A and B fruit accounted for over 80% of the total number of fruits, so type A and B fruits were selected. The seed experiments were repeated for three times, with 30 fruits per treatment for each fruit type in the four species. The seeds were placed in 9-cm-diameter Petri dishes and covered with sand (with a thickness of about 0.5 cm). The sand that came from the top of the dune in the study area was treated to destroy seed bank with a high temperature of 105 °C for 8 hours before germination experiment. The number of emerged seedlings was monitored every 24 hours and germinated seeds were counted and removed from the dishes. Total germination percentage in each treatment was calculated by Formula one after incubation for 21 days. To describe concentration degree and rate of fruit germination in different treatments, and highlight the differences among different treatments, the following formula was applied:

2 $$\begin{array}{ccc}{\rm{Germination}}\,{\rm{rate}} & = & {\rm{Totalgermination}}\,{\rm{percentage}}/{\rm{The}}\,{\rm{days}}\,{\rm{taken}}\\  &  & {\rm{for}}\,{\rm{germination}}\,{\rm{percentages}}\,{\rm{to}}\,{\rm{reached}}\,50 \% (day)\times 100 \% \end{array}$$

### Characteristics of soil seed banks

We collected fresh fruits of the four species from the natural populations. The exact number of the different fruit types was recorded for each species, and they were placed in a nylon bag, which was buried 1 cm below the soil surface under the dead parent plant in October and November 2012 for each species in each plot (repeated three times). In next autumn, the bag was exhumed to count the number of seeds that had germinated in the field. Non-germinated seeds were transported to the laboratory for seed viability testing by staining with tetrazolium.

Soil samples were taken in natural populations from 48 quadrats in two different areas: bare land with no cover of vegetation, and land covered by vegetation. Before seed maturation, all soil in this study was sampled at 10 × 10 × 5 cm (length × width × depth) in October 2013. There were 96 soil seed-bank samples placed in sealed plastic bags and transported to the laboratory. Because we were primarily interested in the characteristics of seed banks of the four species, we quantified soil seed densities via seedling emergence in indoor condition and used mesh screens to sieve the seeds. Ninety-six soil samples were spread in 20 × 20 cm (length × width) plastic plates and sufficient water was provided regularly for seed germination in indoor condition. Any emerging seedlings in the plastic plates were removed and tallied. Meanwhile, soil samples were air-dried after seed germination, being sieved through a 0.8-mm mesh screen and sorted into species. The remaining seeds in the sample were recorded. Finally, non-germinated seeds were transported to the laboratory for seed viability tested by staining with tetrazolium.

### Statistical analyses

Data are expressed as means ± s.e. One-way analysis of variance (ANOVA) was used to identify significant differences among fruit types (A, B, C and D) for fruit diameter, hundred-fruit mass and germination percentage under variable temperature. Fruit type and temperature were used as a fixed factor for “effect of temperature on germination” experiment, and soil moisture content and temperature were used as a fixed factor for “effect of soil moisture content and temperature on germination” experiment. The effects of fruit type and temperature on germination percentage were analyzed with two-way ANOVA. Two-way ANOVA was used to determine the effects of soil moisture content, temperature and their interaction on germination percentage and germination rate of type A, B fruits.

To study germination percentage of different fruit types for each species in natural environment, the number of germinated seeds in the nylon bags was counted and the germination percentage of the different fruit types for each plot was calculated. The germination percentage of different fruit types of the four species was determined by the mean value of all plots. The natural germination percentage of four species among fruit types was tested using One-way ANOVA at 95% confidence interval.

If ANOVA indicated significant differences, LSD multiple range test were used to tesify if the differences (*p* < 0.05) between fruit types or treatments were significant. Analyses were conducted using the software SPSS 20.0 (SPSS Inc., Chicago, IL, USA). Origin 8.6 (Origin Lab, USA) was used as the mapping software.

## Electronic supplementary material

Supplemental files

## References

[1] Kos M, Poschlod P (2010). Why wait? Trait and habitat correlates of variation in germination speed among Kalahari annuals. Oecologia.

[2] Akiyama R, Agren J (2013). Conflicting selection on the timing of germination in a natural population of *Arabidopsis thaliana*. J. Evol. Biol..

[3] Lu JJ, Tan DY, Baskin CC, Baskin JM (2016). Effects of germination season on life history traits and on transgenerational plasticity in seed dormancy in a cold desert annual. Sci. Rep..

[4] Kimball S, Angert AL, Huxman TE, Venable DL (2011). Differences in the timing of germination and reproduction relate to growth physiology and population dynamics of Sonoran Desert winter annuals. Am. J. Bot..

[5] Tozzi E, Van Acker CV (2014). Effects of Seedling Emergence Timing on the Population Dynamics of Horseweed (*Conyza canadensis var*. *canadensis*). Weed Sci..

[6] Huxman TE (2013). Understanding past, contemporary, and future dynamics of plants, populations, and communities using Sonoran Desert winter annuals. Am. J. Bot..

[7] Huang ZY, Liu SS, Bradford KJ, Huxman TE, Venable DL (2016). The contribution of germination functional traits to population dynamics of a desert plant community. Ecology.

[8] Tielbörger K, Petru M (2012). F., X. & Lampei, C. Bet-hedging germination in annual plants: a sound empirical test of the theoretical foundations. Oikos.

[9] Volis S (2012). Demographic consequences of delayed germination in two annual grasses from two locations of contrasting aridity. Perspect. Plant Ecol..

[10] Gremer JR, Kimball S, Venable DL (2016). Within-and among-year germination in Sonoran Desert winter annuals: bet hedging and predictive germination in a variable environment. Ecol. Lett..

[11] Harper, J. L. *Population Biology of Plants*. (Academic Press, London, 1977).

[12] Westoby M (1981). How diversified seed germination behavior is selected?. Am. Nat..

[13] Fenner, M. *Seed Ecology*. (Chapman and Hall, London, 1985).

[14] Baskin JM, Baskin CC (2004). A classification system for seed dormancy. Seed Sci. Res..

[15] Willis CG (2014). The evolution of seed dormancy: environmental cues, evolutionary hubs, and diversification of the seed plants. New Phytol..

[16] Huang ZY, Ölçerfootitt H, Footitt S, Finchsavage WE (2015). Seed dormancy is a dynamic state: variable responses to pre-andpost-shedding environmental signals in seeds of contrasting arabidopsis ecotypes. Seed Sci. Res..

[17] Wijayratne UC, Pyke DA (2012). Burial increases seed longevity of two *Artemisia tridentata* (Asteraceae) subspecies. Am. J. Bot..

[18] Ooi MKJ (2012). Seed bank persistence and climate change. Seed Sci. Res..

[19] Donohue K (2009). Completing the cycle: maternal effects as the missing link in plant life histories. Philos. T. R. Soc. B. Biol. Sci..

[20] Segura F, Vicente MJ, Franco JA, Martínez-Sánchez JJ (2015). Effects of maternal environmental factors on physical dormancy of *Astragalus nitidifloru*sseeds (Fabaceae), a critically endangered species of SE Spain. Flora.

[21] Venable D, Levin D (1985). Ecology of achene dimorphism in *Heterotheca latifolia*: I. Achene structure, germination and dispersal. J. Ecol..

[22] Mandák B, Holmanová Š (2004). The effect of fruit age on seed germinability of a heterocarpic species. Atriplex sagittata. Plant Biol..

[23] Yao SX, Lan HY, Zhang F (2010). C.Variation of seed heteromorphism in *Chenopodium album* and the effect of salinity stress on the descendants. Ann. Bot..

[24] Liu, H. F., Liu, T., Luo, N., Chen, Z. X. & Liu, Z. C. *Salsola affinis* heterocarpy and size-dependent spatial variation. *J*. *Shihezi Univ*. (*Nat*. *Sci*.) **31**, 729–735 (In Chinese with English abstract) (2013).

[25] Lu JJ, Tan DY, Baskin JM, Baskin CC (2014). Germination season and watering regime, but not seed morph, affect life history traits in a cold desert diaspore-heteromorphic annual. Plos One.

[26] Volis S (2016). Seed heteromorphism in *Triticum dicoccoides*: association between seed positions within a dispersal unit and dormancy. Oecologia.

[27] Venable DL (1985). The evolutionary ecology of seed heteromorphism. Am. Nat..

[28] Imbert E (2002). Ecological consequences and ontogeny of seed heteromorphism. Perspect. Plant Ecol..

[29] Lu, A. M. *Geography of family and genera about seed plant*. 270–286 (in Chinese) (Science Press, Beijing, 1999).

[30] Mao Z, Zhang D (1994). The Conspectus of Ephemeral Flora in Northern Xinjiang. Arid Zone Res..

[31] Huang JH (2005). Geographical Distribution of *Salsola* L. in China. Arid Land Geogr..

[32] Luo N, Liu T, Liu HF, Liu ZC, Tang JG (2014). Difference of Seed Germination Characteristics between *Salsola affinis* C.A.Mey and *Salsola brachiata* Pall. in Junggar Basin. J. Shihezi Univ. (Nat. Sci.).

[33] Yang F (2015). Effects of germination time on seed morph ratio in a seed-dimorphic species and possible ecological significance. Ann. Bot..

[34] Grime JP (1977). Evidence for the existence of three primary strategies in plants and its relevance to ecological and evolutionary theory. Am. Nat..

[35] Crawly, M. J. *Plant Ecology*. 97-185 (Blackwell Scientific Publications, London, 1986).

[36] Schwinning S, Sala OE, Loik ME, Ehleringer JR (2004). Thresholds, memory, and seasonality: understanding pulse dynamics in arid/semi-arid ecosystems. Oecologia.

[37] Knapp AK (2008). Consequences of more extreme precipitation regimes for terrestrial ecosystems. Bioscience..

[38] Simons AM (2002). The continuity of microevolution and macroevolution. J. Evolution. Biol..

[39] Slatkin M (1974). Hedging one’s evolutionary bets. Nature.

[40] Gremer JR, Venable DL (2014). Bet hedging in desert winter annual plants: optimal germination strategies in a variable environment. Ecol. Lett..

[41] Philipupillai J, Ungar IA (1984). The Effect of Seed Dimorphism on the Germination and Survival of *Salicornia europaea* L. Populations. Am. J. Bot..

[42] Baskin JM, Lu JJ, Baskin CC, Tan DY, Wang L (2014). Diaspore dispersal ability and degree of dormancy in heteromorphic species of cold deserts of northwest China: A review. Perspect. Plant Ecol..

[43] Wei Y, Dong M, Huang ZY (2007). Seed polymorphism, dormancy and germination of *Salsola affinis* (Chenopodiaceae), a dominant desert annual inhabiting the Junggar Basin of Xinjiang, China. Aust. J. Bot..

[44] Li KL (2012). Seed polymorphism and germination of *Salsola korshinskyi* Drob. J. Shihezi Univ. (Nat. Sci.).

[45] Wang HF, Wei Y, Huang ZY (2007). Seed polymorphism and germination behavior of *Salsola bracchita*, a dominant desert annual inhabiting Junggar Basin of Xinjiang, China. J. Plant Ecol..

[46] Zheng XQ, Zheng XJ, Li Y (2012). Distribution and change of different precipitation pulse sizes in the southern marginal zone of the Junggar Basin, China. Arid Zone Res..

[47] Wertis BA, Ungar IA (1986). Seed Demography and Seedling Survival in a Population of *Atriplex triangularis* Willd. Am. Midl. Nat..

[48] Li, J. F. Climate of Xingjiang. 97–107 (ChinaMeteorological Press, Beijing, 1991) (in Chinese).

[49] Commissione Redactorum Florae Xinjiangensis. *Flora Xinjiangensis*. (Xinjiang Sciences & Technology & Hygiene Publishing House, Urümqi, 1994). (In Chinese).

[50] Pan XL, Zhang HD (1996). Research on the Characteristics of Vegetation and the Forming of Flora in Zhungeer Basin, Xinjiang. Supplement J. Sun Yatsen Univ..

[51] Hu ZY, Hu Q, Zhang C, Chen X, Li QX (2016). Evaluation of reanalysis, spatially interpolated and satellite remotely sensed precipitation data sets in central Asia. J. Geophys. Res..

[52] Berrisford, P. *et al*. The ERA-interim archive. *Era Report* (2009).

