# Supplementary material for: Germination heterochrony in annual plants of Salsola L.: an effective survival strategy in changing environments
Source: Sci Rep. 2018 Apr 26;8:6576. doi: 10.1038/s41598-018-23319-0 (PMC5920052; doi:10.1038/s41598-018-23319-0)

**Germination heterochrony in annual plants of *Salsola* L.: an effective survival strategy in changing environments**

**Hua F. Liu^1^, Tong Liu^1^****^*^, Zhi Q. Han^2^, Ning Luo^1^, Zun C. Liu^1^, Xiao R. Hao^1^**

^1^ *College of Life Sciences,* *Shihezi University, Shihezi 832003, China,* ^2^ *College of Science, Shihezi University, Shihezi 832003, China*

**^*^** *For correspondence author. E-mail* [*betula@126.com*](mailto:betula@126.com)

*Telephone number +86 13579751189*

**Supplemental figure**

1. **The fruit types of four *Salsola* L. species (**The following image shows the typical type of fruit, and the number of fruit types of species was different among plots)


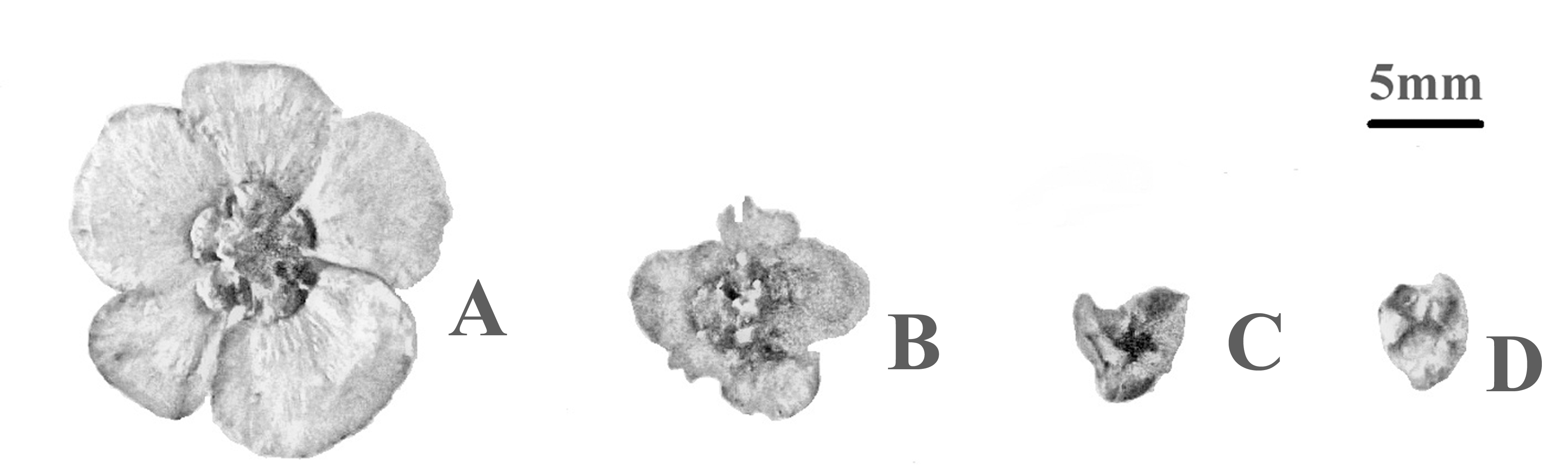


***Salsola affinis***


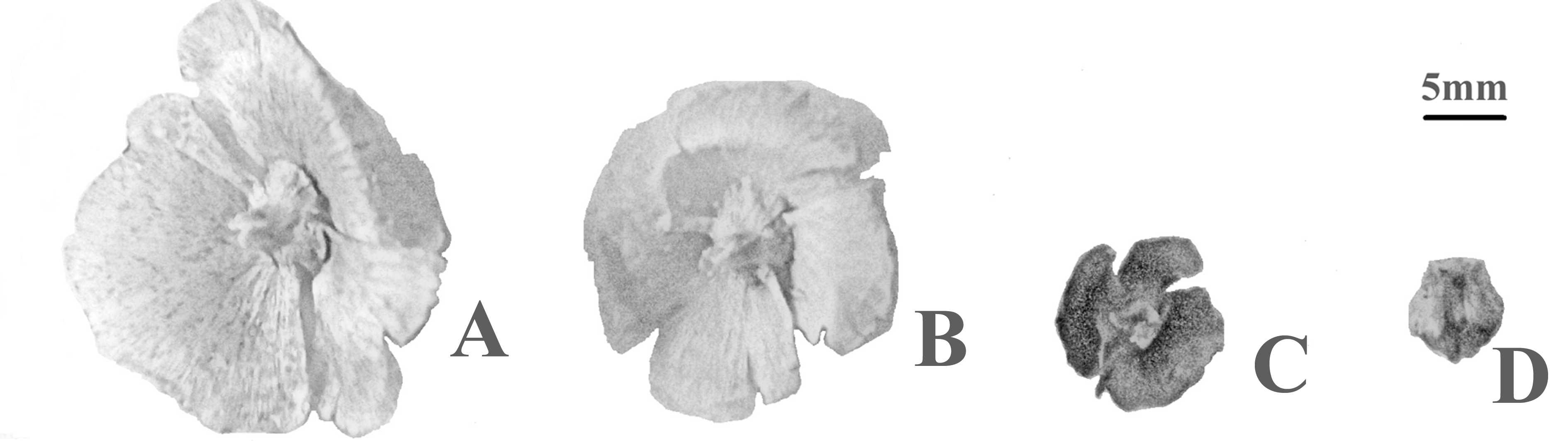


***Salsola korshinskyi***


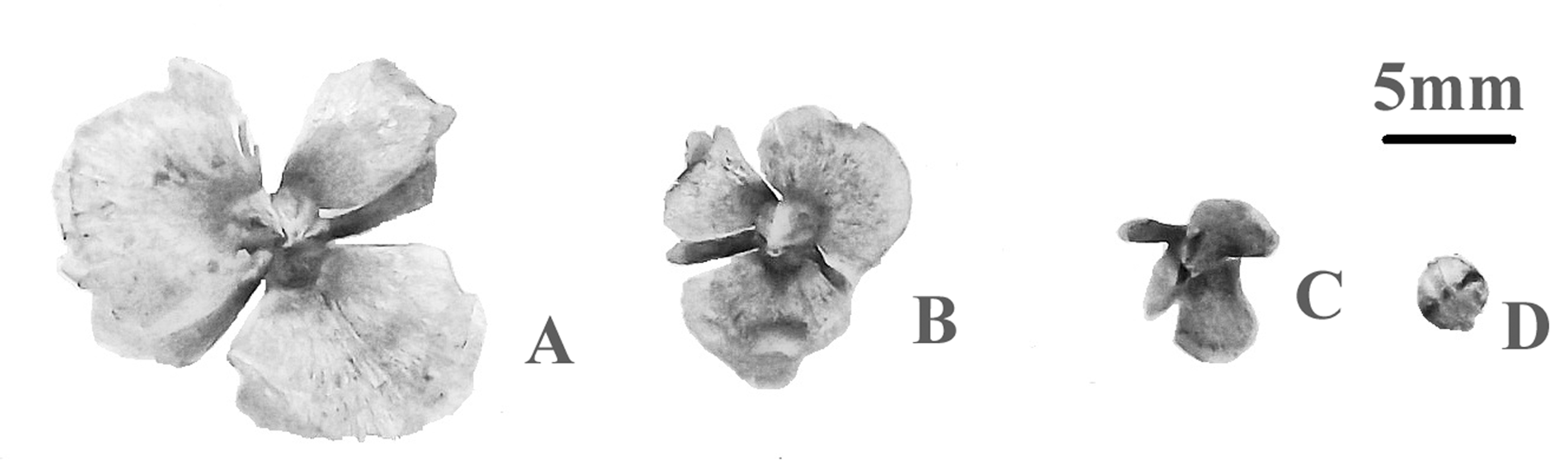


***Salsola nitraria***


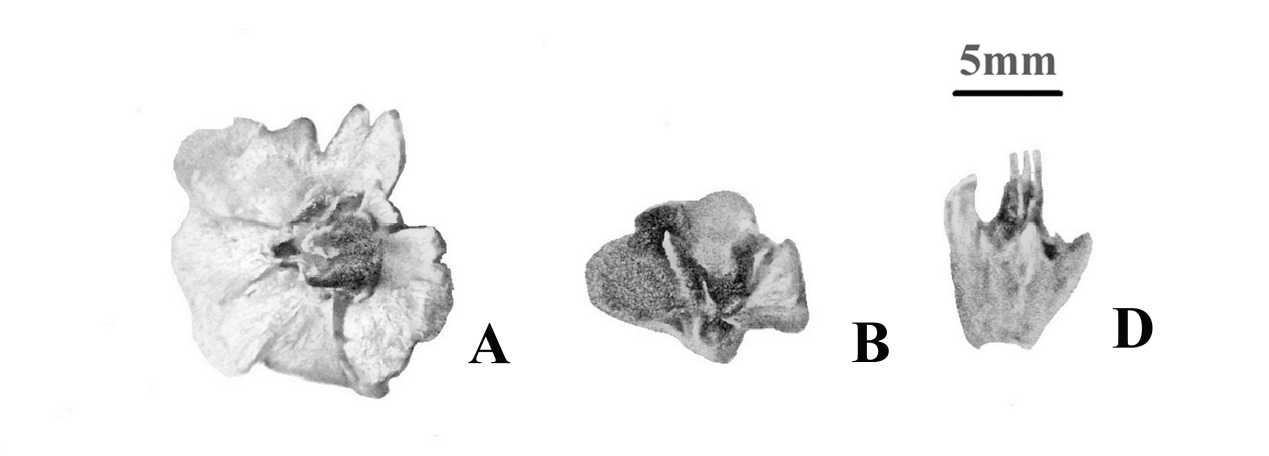


***Salsola brachiata***

1. **Growth difference of individuals that germinated in spring (left) and autumn (right) on fruit maturity stage**


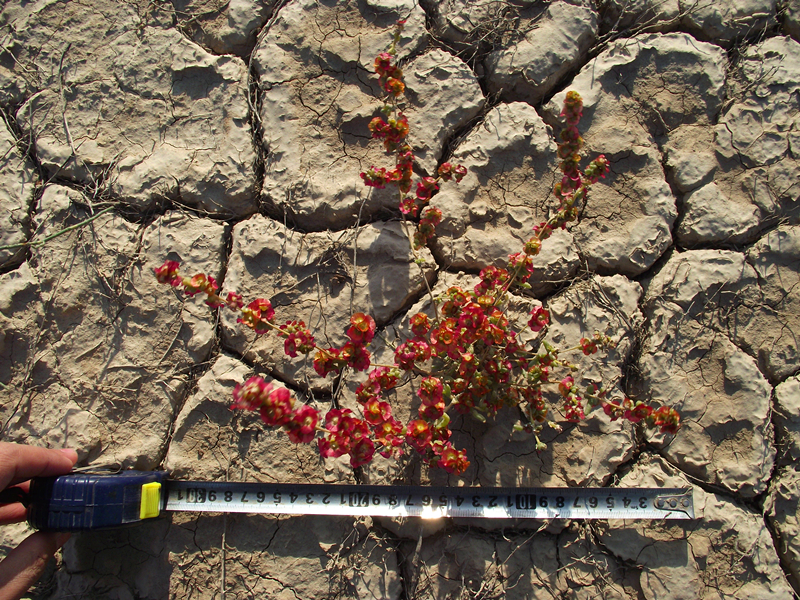

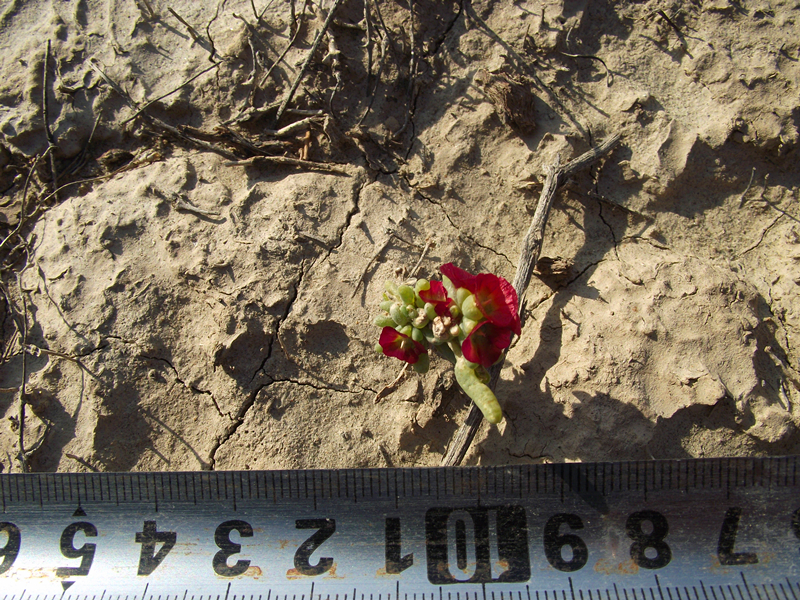

Supplement: Supplementary file 1 — Supplemental files [file 41598_2018_23319_MOESM1_ESM.doc]
